# Rescue of HIV-1 Release by Targeting Widely Divergent NEDD4-Type Ubiquitin Ligases and Isolated Catalytic HECT Domains to Gag

**DOI:** 10.1371/journal.ppat.1001107

**Published:** 2010-09-16

**Authors:** Eric R. Weiss, Elena Popova, Hikaru Yamanaka, Hyung Cheol Kim, Jon M. Huibregtse, Heinrich Göttlinger

**Affiliations:** 1 Program in Gene Function and Expression, Program in Molecular Medicine, University of Massachusetts Medical School, Worcester, Massachusetts, United States of America; 2 Institute for Cellular and Molecular Biology, Section of Molecular Genetics and Microbiology, University of Texas at Austin, Austin, Texas, United States of America; Northwestern University, United States of America

## Abstract

Retroviruses engage the ESCRT pathway through late assembly (L) domains in Gag to promote virus release. HIV-1 uses a PTAP motif as its primary L domain, which interacts with the ESCRT-I component Tsg101. In contrast, certain other retroviruses primarily use PPxY-type L domains, which constitute ligands for NEDD4-type ubiquitin ligases. Surprisingly, although HIV-1 Gag lacks PPxY motifs, the release of HIV-1 L domain mutants is potently enhanced by ectopic NEDD4-2s, a native isoform with a naturally truncated C2 domain that appears to account for the residual titer of L domain-defective HIV-1. The reason for the unique potency of the NEDD4-2s isoform has remained unclear. We now show that the naturally truncated C2 domain of NEDD4-2s functions as an autonomous Gag-targeting module that can be functionally replaced by the unrelated Gag-binding protein cyclophilin A (CypA). The residual C2 domain of NEDD4-2s was sufficient to transfer the ability to stimulate HIV-1 budding to other NEDD4 family members, including the yeast homologue Rsp5, and even to isolated catalytic HECT domains. The isolated catalytic domain of NEDD4-2s also efficiently promoted HIV-1 budding when targeted to Gag via CypA. We conclude that the regions typically required for substrate recognition by HECT ubiquitin ligases are all dispensable to stimulate HIV-1 release, implying that the relevant target for ubiquitination is Gag itself or can be recognized by divergent isolated HECT domains. However, the mere ability to ubiquitinate Gag was not sufficient to stimulate HIV-1 budding. Rather, our results indicate that the synthesis of K63-linked ubiquitin chains is critical for ubiquitin ligase-mediated virus release.

## Author Summary

To promote its escape from cells, HIV-1 hijacks cellular budding machinery through so-called L domains in its structural Gag protein. However, HIV-1 lacks a type of L domain that recruits NEDD4 ubiquitin ligases, a family of cellular enzymes that attach one or more copies of a small protein called ubiquitin to other proteins. Surprisingly, one NEDD4 family member, which is known as NEDD4-2s and stands out because its membrane-binding domain is uniquely truncated, can nevertheless potently stimulate HIV-1 release. Our study reveals that NEDD4-2s can do this because its altered membrane-binding domain allows it to associate with HIV-1 Gag. Remarkably, when tagged with the altered membrane-binding domain of NEDD4-2s, even a distantly related yeast protein becomes capable of stimulating the release of HIV-1. We also show that only the portion of NEDD4-2s that acts as an enzyme is required when targeted to HIV-1 Gag in an alternative manner. Taken together, our findings indicate that it is not simply the ability to attach ubiquitin to Gag, but rather the ability to form a particular type of ubiquitin chain in the immediate vicinity of Gag, that is critical to stimulate virus release.

## Introduction

Retroviruses such as HIV-1 usurp the cellular Endosomal Sorting Complex Required for Transport (ESCRT) machinery to promote the detachment of infectious progeny virions from the plasma membrane [1], [2], [3], [4], [5]. The ESCRT machinery functions in membrane invagination and fission, and was originally identified based on its requirement for the delivery of ubiquitin-tagged membrane proteins into multivesicular endosomes [6], [7]. This process involves the ESCRT-dependent abscission of cellular vesicles from the limiting membrane of endosomes into their lumen, which leads to the formation of multivesicular bodies (MVB) [8], [9]. In addition to its role in MVB biogenesis, the ESCRT machinery is required for midbody abscission during the terminal stage of cytokinesis [10], [11]. Notably, the formation of endosomal vesicles, the separation of daughter cells, and retroviral budding are topologically equivalent events. The ESCRT machinery consists of five heteromeric complexes known as the ESCRT 0-III and VPS4 complexes, and accessory components such as ALIX [6], [8], [12].

Retroviruses recruit the ESCRT machinery through so-called late assembly (L) domains in Gag, the viral polyprotein that drives particle assembly and release [13], [14]. Subsequent to the formation of an immature particle, Gag is cleaved by a virally encoded protease to yield the internal structural components of the mature virion, including matrix (MA), capsid (CA), and nucleocapsid (NC). In addition to these Gag components, which are common to all ortho-retroviruses, HIV-1 Gag possesses a C-terminal p6 domain that harbors two types of L domains. One of these consists of a conserved PTAP motif that functions as the primary HIV-1 L domain and binds to ESCRT-I component Tsg101 [15], [16], [17], [18], [19], [20]. A second L domain in HIV-1 p6 is of the LYPx_n_L-type, binds to the V domain of ALIX, and has an auxiliary role [21], [22], [23], [24], [25]. In contrast to HIV-1, the non-primate lentivirus equine infectious anemia virus engages the ESCRT pathway exclusively via ALIX [21], [22], [23]. Ortho-retroviruses other than lentiviruses primarily use PPxY-type L domains, which constitute ligands for the WW domains of NEDD4 family E3 ubiquitin ligases [4], [13], [14]. Regardless of the type of L domain used, retroviral budding in general is strongly inhibited by dominant-negative versions of ESCRT-III components or of VPS4 [18], [22], [23], [24], [26], [27], [28], indicating that the function of all L domains depends on an intact ESCRT pathway. However, how PPxY-type L domains ultimately engage the ESCRT pathway is not yet understood.

The profound release and infectivity defects of HIV-1 PTAP L domain mutants can be largely corrected by increasing the cellular expression levels of ALIX [21], [29]. This effect of ALIX depends on the LYPx_n_L-type L domain in HIV-1 p6 as expected, and also on the NC domain of Gag, which binds to a different region of ALIX [30], [31], [32]. ALIX interacts directly with components of the ESCRT-III complex [22], [23], [24], [33], [34], which is thought to carry out membrane scission reactions at bud necks [35]. Remarkably, despite the absence of a PPxY-type L domain from HIV-1 Gag, the budding, processing, and infectivity defects of HIV-1 L domain mutants can also be potently corrected through the overexpression of the NEDD4 family ubiquitin ligase NEDD4-2s [36], [37]. In contrast to ALIX, NEDD4-2s is capable of rescuing HIV-1 release in the absence of all known L domains [36], [37]. To stimulate HIV-1 budding, NEDD4-2s must be catalytically active, implying that the conjugation of ubiquitin to a viral or cellular substrate is required [36], [37]. Importantly, an siRNA directed against all NEDD4-2 isoforms substantially reduced the already low level of particle production by HIV-1_ΔPTAPP_ [37], indicating that NEDD4-2s accounts for a large portion of the residual release that is often observed in the absence of a Tsg101 binding site.

NEDD4 family ubiquitin ligases have a common modular architecture, with an N-terminal C2 domain involved in membrane binding, multiple substrate-binding WW domains, and a C-terminal HECT domain with intrinsic catalytic activity [38]. NEDD4-2s is a native, common isoform of human NEDD4-2 that lacks most of the C2 domain due to alternative exon usage [39]. However, the portion of the C2 domain that remains is essential for the ability of NEDD4-2s to stimulate HIV-1 release, indicating that it constitutes an important functional domain of the protein [36]. In our hands, NEDD4-2s was unique among several NEDD4 family members in its ability to rescue HIV-1_ΔPTAPP_, with even full-length NEDD4-2 showing only minimal activity [36]. In contrast, in a study by Sundquist and colleagues, full-length NEDD4-2 also stimulated HIV-1_ΔPTAP_ release and infectivity [37]. However, in agreement with our results [36], only the NEDD4-2s isoform reduced the accumulation of Gag processing intermediates, indicating that NEDD4-2s is uniquely potent [37].

In the present study, we show that the residual C2 domain of NEDD4-2s constitutes an autonomous HIV-1 Gag-targeting module that can be functionally replaced by the unrelated HIV-1 Gag-binding protein cyclophilin A (CypA). Remarkably, CypA could also substitute for the entire substrate-recognition domain of NEDD4-2s. When targeted to Gag via the C2 domain remnant of NEDD4-2s, other NEDD4-type ubiquitin ligases, including yeast Rsp5, and a subset of isolated HECT domains acquired the ability to rescue HIV-1 budding. These observations imply that divergent isolated HECT domains are either capable of recognizing a functionally relevant substrate when targeted to Gag, or can engage the ESCRT pathway themselves upon autoubiquitination. Our results also indicate that the synthesis of K63-linked ubiquitin chains by NEDD4 family members is critical for the ability to stimulate virus budding.

## Results

### Targeting of the catalytic HECT domain of NEDD4-2 to Gag is sufficient to rescue HIV-1_ΔPTAPP_

**Figure 1:**
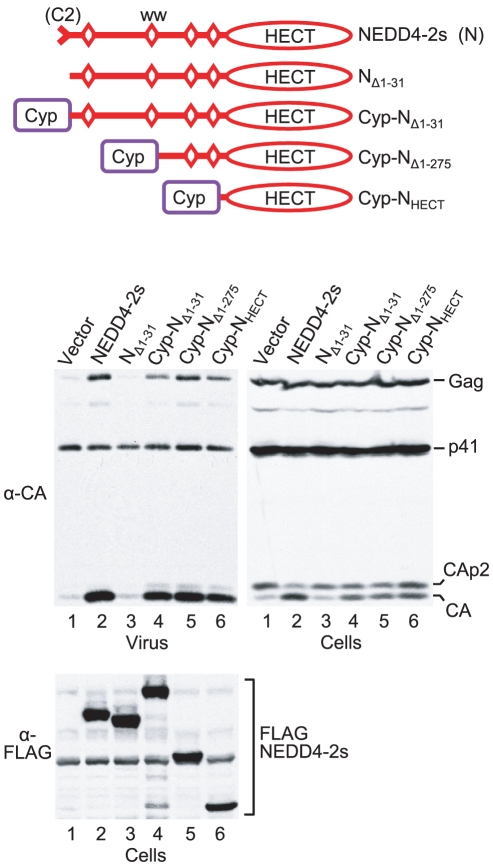
The isolated HECT domain of NEDD4-2s rescues HIV-1_ΔPTAPP_ when targeted to Gag. 293T cells were transfected with HXBH10_ΔPTAPP_ (1 µg) and empty vector, or vectors (2 µg each) expressing FLAG-tagged WT NEDD4-2s, a version lacking the residual C2 domain (N_Δ1-31_), or versions that have portions of NEDD4-2s replaced by the HIV-1 Gag-binding protein CypA as illustrated. Virion pellets and the cell lysates were analyzed by Western blotting to detect Gag, Gag cleavage products, and FLAG-tagged proteins as indicated.

We previously showed that the residual C2 domain of NEDD4-2s is required for its ability to rescue HIV-1_ΔPTAPP_ [36]. Since our results suggested that the residual C2 domain is needed for NEDD4-2s to associate with Gag [36], we reasoned that it may be possible to functionally replace the residual C2 domain with an unrelated sequence known to bind HIV-1 Gag. To test this hypothesis, we replaced the residual C2 domain of NEDD4-2s (residues 1 to 31) with human cyclophilin A (CypA) followed by HA and FLAG epitopes, yielding Cyp-N_Δ1-31_ (Fig. 1). CypA is a 165 amino acid cytosolic protein that binds to the CA domain of HIV-1 Gag [40], [41]. Its presence at the N-terminus of Cyp-N_Δ1-31_ was thus expected to target the fusion protein to assembling HIV-1 virions.

Consistent with our previous results [36], we observed a 32-fold increase in the release of particle-associated CA when 293T cells were cotransfected with HIV-1_ΔPTAPP_ and a vector expressing FLAG-tagged NEDD4-2s (Fig. 1). NEDD4-2s also corrected the Gag cleavage defect of HIV-1_ΔPTAPP_, which leads to the accumulation of CA-p2 at the expense of mature CA in virus-producing cells. Although the mechanistic basis for this cleavage defect remains unknown, impaired processing at the CA-p2 site is considered a hallmark of late assembly defects. As expected, a version of NEDD4-2s that had the residual C2 domain precisely deleted (here called N_Δ1-31_) was completely inactive, again confirming earlier results [36]. In contrast, Cyp-N_Δ1-31_ was nearly as active as WT NEDD4-2s, and stimulated the release of particulate CA approximately 25-fold. Furthermore, Cyp-N_Δ1-31_ partially corrected the Gag processing defect of the Tsg101 binding site mutant (Fig. 1). Thus, the presence of CypA in place of the residual C2 domain of NEDD4-2s restored activity in the ΔPTAPP rescue assay.

The residual C2 domain of NEDD4-2s is separated from the catalytic HECT domain by an approximately 420-amino-acid region that harbors four WW domains involved in substrate binding (Fig. 1). To examine whether this substrate-binding region is required for the rescue of HIV-1_ΔPTAPP_, we fused CypA followed by HA and FLAG epitopes to truncated versions of NEDD4-2s that lacked either two (N _Δ1-275_) or all four of the WW domains, in effect leaving only the HECT domain in place (N_HECT_). As shown in Fig. 1, both chimeric constructs were highly active in the ΔPTAPP rescue assay. Specifically, Cyp-N_Δ1-275_ and Cyp-N_HECT_ stimulated the release of particle-associated CA by 30- and 20-fold, respectively. Of note, identical NEDD4-2s truncation mutants that lack CypA at the N-terminus exhibited no activity in this assay [36]. We conclude that the targeting of the isolated catalytic HECT domain of NEDD4-2s to Gag via CypA is sufficient to rescue HIV-1 budding defects.

### Transfer of the residual C2 domain of NEDD4-2s confers the ability to rescue HIV-1 release to a yeast homologue

**Figure 2:**
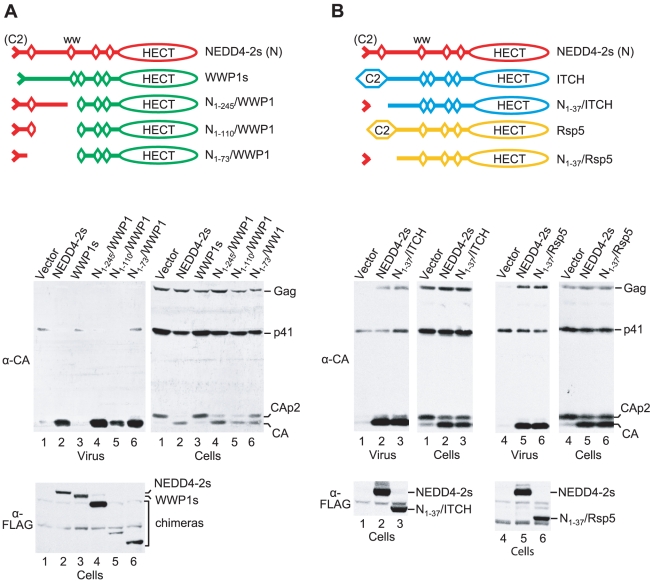
The residual C2 domain of NEDD4-2s is sufficient to transfer the ability to rescue HIV-1_ΔPTAPP_. (**A**) The transfer of NEDD4-2s sequences upstream of the first WW domain to WWP1 is sufficient to confer activity in the ΔPTAPP rescue assay. (**B**) Human ITCH and yeast Rsp5 potently rescue HXBH10_ΔPTAPP_ when their C2 domain is replaced by the residual C2 domain of NEDD4-2s. 293T cells were transfected with HXBH10_ΔPTAPP_ (1 µg) and vectors (2 µg each) expressing the indicated FLAG-tagged parental or chimeric ubiquitin ligase constructs, or the empty vector. Virion pellets and the cell lysates were analyzed by Western blotting to detect Gag, Gag cleavage products, and FLAG-tagged proteins as indicated.

In our previous study, the overexpression of the NEDD4 family ubiquitin ligases NEDD4-1, WWP1, WWP2, or ITCH had no significant effect on the release of HIV-1_ΔPTAPP_ [36]. Since all these proteins possess an intact C2 domain, we had also generated a variant of WWP1 that lacks the exact portion of the C2 domain which is naturally absent from NEDD4-2s. The resulting WWP1s mutant remained inactive in the ΔPTAPP rescue assay, indicating that the robust activity of NEDD4-2s is not simply due to its naturally truncated C2 domain [36]. To determine what region of NEDD4-2s accounts for its unique ability to rescue HIV-1 budding, we generated FLAG-tagged chimeras between NEDD4-2s and WWP1 as illustrated in Fig. 2A. Interestingly, whereas WWP1s exhibited no activity as previously reported [36], a version that included the first 245 residues of NEDD4-2s (N_1-245_/WWP1) was at least as active as WT NEDD4-2s in rescuing particle production and Gag processing by HIV-1_ΔPTAPP_ (Fig. 2A). Quantitation of the amount of virion-associated CA indicated that NEDD4-2s increased mature particle production 25-fold in this experiment, whereas N_1-245_/WWP1 had a 43-fold effect. The N-terminal 245 residues of NEDD4-2s include the truncated C2 domain, WW domain 1, and the relatively long intervening segment that separates WW domains 1 and 2. A chimeric molecule that lacked this intervening segment (N_1-110_/WWP1) increased mature particle production by HIV-1_ΔPTAPP_ almost 20-fold, despite being poorly expressed (Fig. 2A). Finally, HIV-1_ΔPTAPP_ released almost 40-fold more mature CA when co-expressed with the N_1-73_/WWP1 chimera (Fig. 2A), demonstrating that only NEDD4-2s sequences upstream of WW domain 1 were required for activity.

The results described above suggested to us that the unique ability of NEDD4-2s to promote HIV-1 budding may be solely determined by its unique residual C2 domain. To test this notion, we examined whether we could confer activity to the NEDD4 family member ITCH by replacing its C2 domain with a 37-amino-acid fragment from NEDD4-2s that harbors its truncated C2 domain [42]. As shown in Fig. 2B, the resulting N_1-37_/ITCH chimera corrected the release and Gag processing defects of HIV-1_ΔPTAPP_ as efficiently as NEDD4-2s itself. Although ITCH was recently reported to potently rescue a murine leukemia virus L domain mutant [43], we previously observed that native ITCH does not rescue HIV-1_ΔPTAPP_ [36]. We thus conclude that the truncated C2 domain of NEDD4-2s is both required and sufficient to transfer the ability to rescue HIV-1 budding.

To determine the generality of these findings, we examined whether it was possible to convert a yeast ubiquitin ligase into a form capable of correcting HIV-1 budding defects. *Saccharomyces cerevisiae* encodes a single essential NEDD4 family ubiquitin ligase, Rsp5. Like human NEDD4 family members, Rsp5 possesses an N-terminal C2 domain, WW domains, and a C-terminal HECT domain. As illustrated in Fig. 2B, we replaced the C2 domain of FLAG-tagged yeast Rsp5 with the N-terminal 37 amino acids of human NEDD4-2s, which yielded the N_1-37_/Rsp5 chimera. Remarkably, when expressed in human 293T cells together with HIV-1_ΔPTAPP_, the N_1-37_/Rsp5 fusion protein rescued particle production and Gag processing by the Tsg101 binding site mutant as potently as Nedd4-2s, despite being expressed at lower levels (Fig. 2B). In contrast, the parental FLAG-Rsp5 construct had only a minor effect on particle production by HIV-1_ΔPTAPP_, and did not noticeably affect the Gag processing defect of HIV-1_ΔPTAPP_ (data not shown). We infer that the residual C2 domain of NEDD4-2s is sufficient to confer the ability to function in HIV-1 budding to widely divergent NEDD4 family members from different species.

### The residual C2 domain of NEDD4-2s confers the ability to associate with HIV-1 Gag

We previously showed that the residual C2 domain of NEDD4-2s is required for its ability to induce Gag ubiquitination, and for its incorporation into VLP [36]. To examine these parameters, we had made use of an HIV-1 Gag construct called Z_WT_, which has NC and p6 replaced by a foreign dimerization domain and efficiently produces VLP in an ESCRT pathway-independent manner [44], [45]. Furthermore, Z_WT_ Gag exhibits less baseline ubiquitination than authentic HIV-1 Gag. These properties of Z_WT_ Gag had allowed us to analyze the effect of NEDD4-2s on the ubiquitination of VLP-associated Gag, and the uptake of NEDD4-2s into VLP, independent of its effect on VLP production [36].

**Figure 3:**
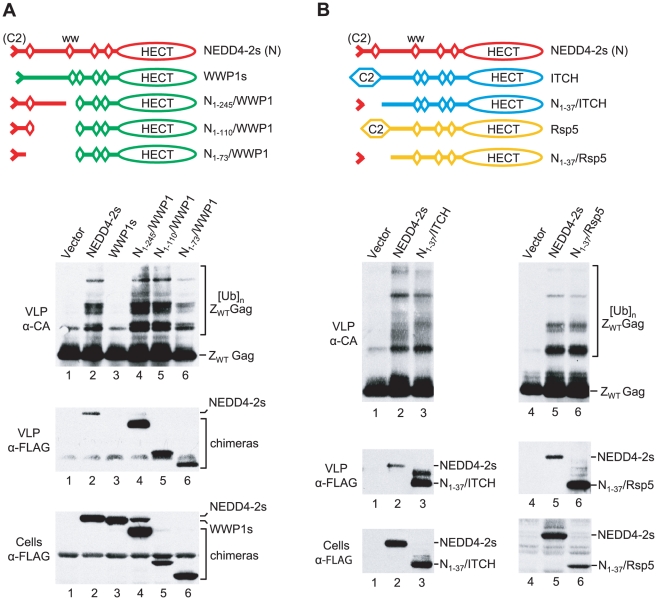
The residual C2 domain of NEDD4-2s functions as a Gag-targeting module. (**A**) NEDD4-2s sequences upstream of the first WW domain are sufficient to induce the ubiquitination of an L domain-independent HIV-1 Gag construct (Z_WT_) by WWP1, and the association of WWP1 with Z_WT_ VLP. (**B**) Human ITCH and yeast Rsp5 ubiquitinate Z_WT_ Gag and associate with Z_WT_ VLP when their C2 domain is replaced by the NEDD4-2s residual C2 domain. 293T cells were transfected with the Z_WT_ Gag construct (2 µg) and vectors (2 µg each) expressing the indicated FLAG-tagged parental or chimeric ubiquitin ligase constructs, or the empty vector. VLP were analyzed by Western blotting with anti-CA to detect unmodified and ubiquitinated versions of Z_WT_ Gag, and with anti-FLAG to detect the incorporation of ubiquitin ligase constructs into VLP. The cell lysates were also examined with anti-FLAG.

We used the same Z_WT_ Gag-based assay system as in our previous study to determine whether chimeric versions of WWP1, ITCH, or Rsp5 are capable of inducing Gag-ubiquitin conjugates. All of the parental and chimeric ubiquitin ligase constructs used in this analysis had N-terminal FLAG tags to allow a comparison of relative expression levels. As previously reported [36], the co-expression of Z_WT_ Gag and NEDD4-2s led to the appearance of additional Gag species in Z_WT_ VLP that migrated slower than the Z_WT_ Gag precursor (Fig. 3A). We have demonstrated that these NEDD4-2s-induced bands represent Gag-ubiquitin conjugates [36]. In contrast, WWP1s, a version of WWP1 that lacks the portion of the C2 domain that is naturally absent in NEDD4-2s [36], did not induce Gag-ubiquitin-conjugates in the same assay (Fig. 3A). Furthermore, in contrast to NEDD4-2s, WWP1s was not incorporated into Z_WT_ VLP, even though both were expressed at comparable levels (Fig. 3A). These findings are in agreement with the lack of activity of WWP1s in the ΔPTAPP rescue assay [36]. Interestingly, the N_1-245_/WWP1, N_1-110_/WWP1, and N_1-73_/WWP1 chimeras, which were active in the ΔPTAPP rescue assay (see Fig. 2A), were all incorporated into Z_WT_ VLP and induced a similar pattern of Gag-ubiquitin conjugates as NEDD4-2s (Fig. 3A).

Similar results as with the WWP1-based chimeras were obtained with N_1-37_/ITCH and N_1-37_/Rsp5 (Fig. 3B). We found that N_1-37_/ITCH and N_1-37_/Rsp5 both induced a very similar extent of Gag ubiquitination as NEDD4-2s. Further, particularly in the case of N_1-37_/Rsp5, the pattern of Gag-ubiquitin conjugates was virtually indistinguishable from that induced by NEDD4-2s. Also, both N_1-37_/ITCH and N_1-37_/Rsp5 were taken up into VLP even more efficiently than NEDD4-2s. Together, these results imply that the residual C2 domain of NEDD4-2s is sufficient to transfer the ability to associate with HIV-1 Gag to other NEDD4 family members, which thereby gain access into VLP and the ability to ubiquitinate Gag.

### Isolated HECT domains differ in their ability to rescue HIV-1 budding if fused to the residual C2 domain of NEDD4-2s

**Figure 4:**
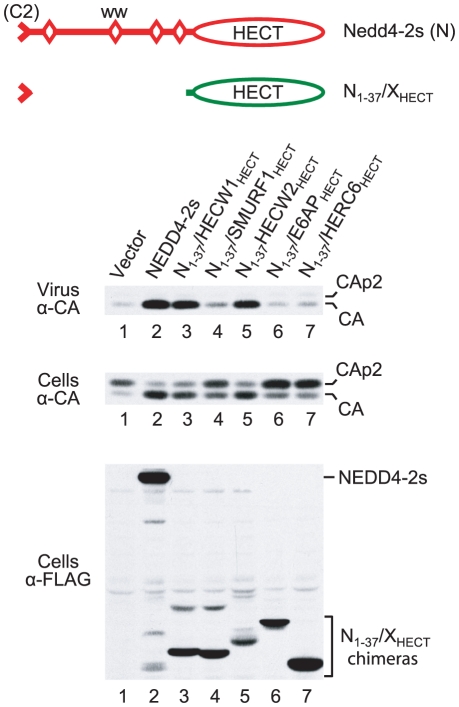
Rescue of HIV-1_ΔPTAPP_ by isolated HECT domains fused to the NEDD4-2s residual C2 domain. 293T cells were transfected with HXBH10_ΔPTAPP_ (0.5 µg) and empty vector, or vectors expressing FLAG-tagged versions of NEDD4-2s (2 µg) or of the indicated fusion proteins (6 µg each). Virion pellets and the cell lysates were analyzed by Western blotting to detect Gag products and FLAG-tagged proteins as indicated.

As described above, we found that the isolated HECT domain of NEDD4-2s is sufficient to rescue HIV-1_ΔPTAPP_ if brought into contact with Gag. Furthermore, our results suggested that the residual C2 domain of NEDD4-2s can serve as a Gag-binding module. We therefore asked whether the isolated HECT domains of other human HECT ubiquitin ligases can promote HIV-1 budding if fused to the residual C2 domain of NEDD4-2s. Among the HECT domains included in this analysis were those of the NEDD4 family members SMURF1, HECW1, and HECW2, which are derived from different ancestral genes than NEDD4-2s, WWP1, or ITCH [46]. Additionally, we included the HECT domains of E6AP and of HERC6, which belong to the SI-HECT and HERC subfamilies of HECT E3s, respectively [38]. All HECT domains were directly appended to a FLAG-tagged version of the residual C2 domain of NEDD4-2s, and the fusion proteins were expressed in 293T cells together with HIV-1_ΔPTAPP_. Two of the HECT domain constructs (N_1-37_/HECW1_HECT_ and N_1-37_/HECW2_HECT_) rescued both particle production and the conversion of CA-p2 to mature CA to a similar extent as full-length NEDD4-2s, which served as a positive control (Fig. 4). In the experiment shown in Fig. 4, NEDD4-2s stimulated particle production 28-fold, and the HECT domain constructs N_1-37_/HECW1_HECT_ and N_1-37_/HECW2_HECT_ enhanced virus release 24- and 20-fold, respectively. On the other hand, equivalent constructs that harbored the HECT domains of SMURF1, E6AP, or HERC6 were poorly active or inactive in the ΔPTAPP rescue assay, even though all were well expressed (Fig. 4).

**Figure 5:**
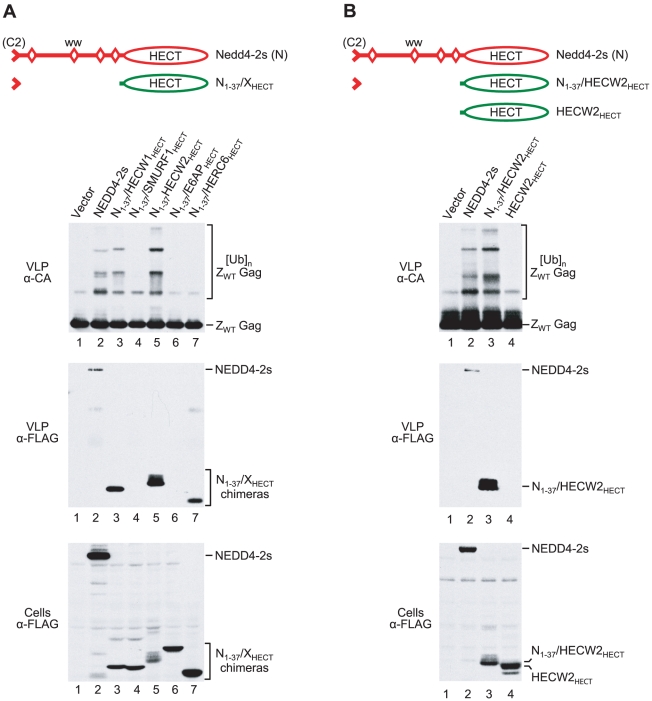
The NEDD4-2s residual C2 domain is sufficient to target some isolated HECT domains to Gag. (**A**) Residual C2 domain/HECT domain fusion proteins differ in their ability to induce the ubiquitination of Z_WT_ Gag, and to associate with Z_WT_ VLP. (**B**) The ubiquitination of Z_WT_ Gag by the isolated HECT domain of HECW2 and its association with Z_WT_ VLP were dependent on the presence of the NEDD4-2s residual C2 domain. 293T cells were transfected with the Z_WT_ Gag construct (2 µg) and empty vector, or vectors expressing FLAG-tagged versions of NEDD4-2s (2 µg) or of the indicated HECT domain constructs (6 µg each).

As shown in Fig. 5A, the two HECT domain constructs that were active in the ΔPTAPP rescue assay (N_1-37_/HECW1_HECT_ and N_1-37_/HECW2_HECT_) induced the ubiquitination of Z_WT_ Gag to a comparable extent as NEDD4-2s. In contrast, the constructs harboring the HECT domains of SMURF1, E6AP, or HERC6 failed to induce Gag ubiquitination. It is possible that the isolated HECT domains of E6AP and HERC6 lacked catalytic activity, since no evidence for autoubiquitination was apparent. Also, HERC6 is an interferon-induced protein that, based on similarity to human HERC5 and mouse HERC6, may be a ligase for ISG15 rather than ubiquitin [47], [48].However, the N_1-37_/SMURF1_HECT_ construct appeared as capable of autoubiquitination as the two HECT domain constructs that efficiently rescued HIV-1 budding (Fig. 5A; also see Fig. 4).

To more directly determine whether the HECT domain constructs were capable of associating with HIV-1 Gag, we also examined their incorporation into Z_WT_ VLP. The two active HECT domain constructs were readily detectable in VLP. If fact, relative to their expression levels, N_1-37_/HECW1_HECT_ and N_1-37_/HECW2_HECT_ were incorporated at 14- and 46-fold higher levels than NEDD4-2s, respectively. In contrast, two of the three inactive constructs (N_1-37_/SMURF1_HECT_ and N_1-37_/E6AP_HECT_) were not incorporated, even though they were expressed as well or better than the constructs that were taken up into VLP. The third inactive construct (N_1-37_/HERC6_HECT_) was incorporated at about 4-fold higher levels than NEDD4-2s, normalized for expression levels (Fig. 5A). The N_1-37_/HECW2_HECT_ construct exhibited the lowest level of expression but the highest level of VLP association, which suggested that the HECT domain of HECW2 in particular contributed to the association with HIV-1 Gag. However, a version of N_1-37_/HECW2_HECT_ that lacked the residual C2 domain of NEDD4-2s did not induce Gag ubiquitination, was not incorporated into VLP, and was inactive in the ΔPTAPP rescue assay (Fig. 5B and data not shown).

Taken together, these results show that the residual C2 domain of NEDD4-2s is sufficient to target a subset of isolated HECT domains to HIV-1 Gag. They also show that the isolated HECT domain of NEDD4-2s is not unique in its ability to rescue HIV-1 budding when targeted to Gag. Rather, this ability is shared by the HECT domains of the two human NEDD4 family members that are overall most divergent from NEDD4-2s.

### Induction of Gag ubiquitination is not sufficient to rescue of HIV-1 budding

**Figure 6:**
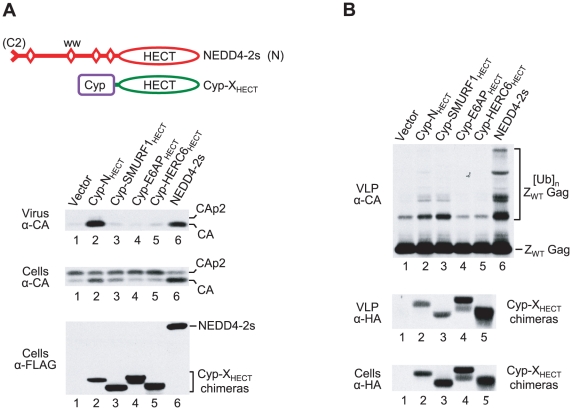
Induction of Gag ubiquitination is not sufficient to rescue of HIV-1 release. (**A**) The isolated HECT domain of NEDD4-2s rescues HXBH10_ΔPTAPP_ when targeted to Gag via CypA (lane 2), whereas the isolated HECT domains of SMURF1, E6AP, or HERC6 do not (lanes 3–5). (**B**) The isolated HECT domains of NEDD4-2s (lane 2) and SMURF1 (lane 3) induced comparable levels of Gag ubiquitination when fused to CypA. 293T cells were transfected with HXBH10_ΔPTAPP_ (0.5 µg) or Z_WT_ (2 µg) and empty vector, or vectors expressing the indicated FLAG/HA-tagged CypA-HECT domain fusion proteins or FLAG-tagged NEDD4-2s (2 µg each). Gag proteins were detected with anti-CA, and the CypA-HECT fusion proteins were detected with anti-FLAG or anti-HA, as indicated.

The apparent inability of the N_1-37_/SMURF1_HECT_ construct to associate with HIV-1 Gag offered a possible explanation for its failure to rescue HIV-1_ΔPTAPP_ and to ubiquitinate Z_WT_ Gag, despite being able to auto-ubiquitinate. We therefore examined the effect of targeting the SMURF1 HECT domain to HIV-1 Gag via CypA. As shown in Fig. 6A, fusing the HECT domains of SMURF1, E6AP, or HERC6 directly to CypA did not confer the ability to rescue HIV-1_ΔPTAPP_. In contrast, the equivalent Cyp-N_HECT_ construct, which instead contains the HECT domain of NEDD4-2s, did rescue nearly as well as WT NEDD4-2s, as expected. Although all CypA-HECT domain fusion proteins were incorporated into Z_WT_ VLP, those containing the HECT domain of E6AP or HERC6 did not ubiquitinate Z_WT_ Gag, consistent with the possibility that these HECT domains are catalytically inactive in isolation (Fig. 6B). Surprisingly, the CypA fusion proteins with the NEDD4-2s and SMURF1 HECT domains induced comparable levels of Gag-ubiquitin conjugates (Fig. 6B), even though only the former was active in the ΔPTAPP rescue assay (Fig. 6A). Moreover, although the CypA fusion protein with the NEDD4-2s HECT domain (Cyp-N_HECT_) and WT NEDD4-2s exhibited comparable activities in the ΔPTAPP rescue assay (Fig. 6A), the former induced a much lower level of Z_WT_ Gag ubiquitination (Fig. 6B). These results demonstrate that the mere ability to ubiquitinate Gag is not sufficient to rescue HIV-1 budding. Furthermore, if the ubiquitination of Gag is necessary to promote budding, then only relatively low levels of Gag ubiquitination are required.

### Rescue of HIV-1 budding correlates with chain type specificity

The lack of correlation between the rescue of HIV-1 budding and the overall levels of Gag ubiquitination led us to investigate the possible role of the type of ubiquitin chain that can be formed. It was recently shown that ITCH, which potently rescues HIV-1 budding when targeted to Gag (Fig. 2B), has a very high preference for the synthesis of K63-linked polyubiquitin chains [49]. However, the replacement of the C lobe of the HECT domain of ITCH by that of E6AP caused a complete switch to K48 chain type specificity [49]. On the other hand, an ITCH-HUWE1 C-lobe chimera produced both relatively short K48 chains and even shorter K63 chains [49]. HUWE1 is a large HECT domain ubiquitin ligase that does not belong to the NEDD4 family, but is relatively closely related in its HECT domain.

**Figure 7:**
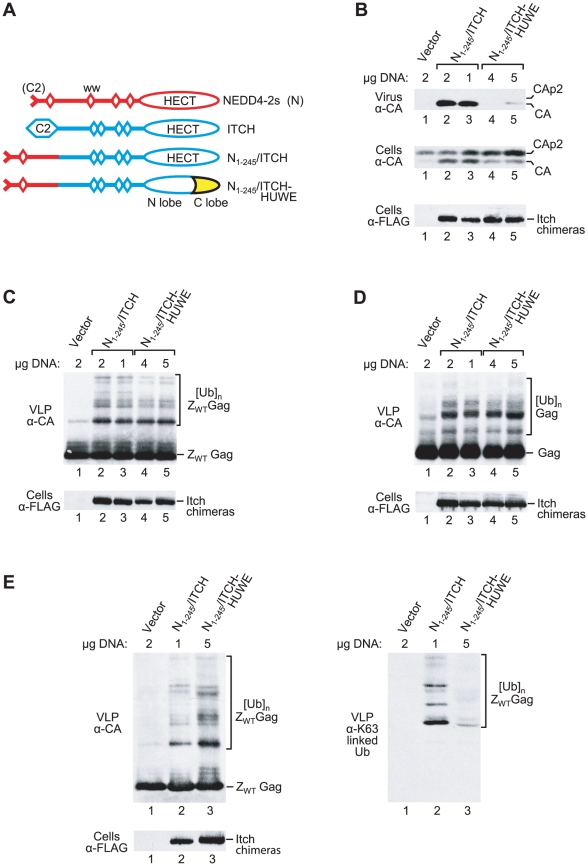
Rescue of HIV-1 release correlates with chain type specificity. (**A**) Schematic illustration of chimeric ubiquitin ligase constructs. The C lobe of HUWE1 is indicated in yellow. (**B to D**) Replacement of the C lobe of the ITCH HECT domain by that of HUWE1 greatly impairs the rescue of HIV-1_ΔPTAPP_ by a NEDD4-2s/ITCH chimera (panel B), but not the overall ubiquitination of VLP-associated Z_WT_ Gag (panel C) or of virus particle-associated authentic Gag expressed by an HIV-1 provirus lacking protease (panel D). Expression vectors for HECT domain constructs were transfected in varying amounts as indicated to achieve comparable expression levels. (**E**) The parental N_1-245_/ITCH construct, in contrast to the N_1-245_/ITCH-HUWE1 HECT domain chimera, induces the attachment of K63-linked chains to Z_WT_ Gag. 293T cells were transfected with the Z_WT_ Gag construct and empty vector, or vectors expressing FLAG-tagged versions of the indicated HECT domain constructs. VLP samples were analyzed by Western blotting with anti-CA to detect unmodified and ubiquitinated versions of Z_WT_ Gag (left panel), and in parallel with Apu3 to detect K63-linked ubiquitin chains conjugated to Z_WT_ Gag (right panel). The cell lysates were also examined with anti-FLAG.

To examine the role of chain type specificity in the rescue of HIV-1 budding, we replaced the C lobe of N_1-245_/ITCH by that of E6AP or HUWE1, as illustrated in Fig. 7A. N_1-245_/ITCH rescues HIV-1_ΔPTAPP_ as potently as N_1-37_/ITCH, and was used here because HECT C lobe chimeras based on this construct were relatively stable (data not shown). The N_1-245_/ITCH E6AP C lobe chimera appeared to have no effect on HIV-1 budding, but was too poorly expressed to yield reliable results (data not shown). In contrast, the N_1-245_/ITCH-HUWE1 C lobe chimera was expressed at higher levels. However, even at expression levels similar to or higher than those obtained with the parental N_1-245_/ITCH construct, N_1-245_/ITCH-HUWE1 had at most a small effect on the release of HIV-1_ΔPTAPP_, whereas N_1-245_/ITCH potently rescued budding (Fig. 7B). Nevertheless, the parental N_1-245_/ITCH construct and N_1-245_/ITCH-HUWE1 induced comparable levels of ubiquitination both of Z_WT_ Gag (Fig. 7C) and of authentic HIV-1 Gag expressed by a proviral protease mutant (Fig. 7D).

Interestingly, the pattern of Z_WT_ Gag ubiquitination seen with N_1-245_/ITCH-HUWE1 differed slightly from that obtained with N_1-245_/ITCH (Fig. 7C), consistent with the possibility that these two proteins catalyze different chain linkages, as previously shown for ITCH-HUWE1 and ITCH in vitro [49]. To examine this possibility directly, we made use of the linkage-specific antibodies Apu3 and HWA4C4, which exhibit high selectivity for K63-linked ubiquitin chains [50], [51]. In the experiment shown in Fig. 7E, N_1-245_/ITCH-HUWE1 was more highly expressed than N_1-245_/ITCH, and induced higher overall levels of Z_WT_ Gag ubiquitination as detected by immunoblotting of Z_WT_ particles with anti-CA antibody (left panel). Nevertheless, when the same samples were examined by Western blotting with the K63-linkage specific Apu3 antibody, only the parental N_1-245_/ITCH construct yielded three prominent bands (right panel). The mobility of these three bands was as expected for Z_WT_ Gag modified with di-, tri-, or tetra-ubiquitin chains. Equivalent results were obtained with HWA4C4, another K63-linkage specific antibody (data not show). We conclude that the chain type specificities of N_1-245_/ITCH and N_1-245_/ITCH-HUWE1 in living cells differ considerably, and that the ability to catalyze K63-linked chains is of critical importance for the rescue of HIV-1 release.

## Discussion

We show here that widely divergent human and yeast ubiquitin ligases of the NEDD4 family, and even a subset of isolated HECT domains, possess the intrinsic ability to function in HIV-1 release. The truncated C2 domain of NEDD4-2s provides a natural Gag-targeting module, which accounts for the unique ability of authentic NEDD4-2s to rescue HIV-1 budding defects. However, other NEDD4 family members, including yeast Rsp5, and in some cases even their isolated catalytic HECT domains, acquire the same ability if targeted to HIV-1 Gag. A common property that is shared by widely divergent NEDD4 family members is the preferential catalysis of K63-linked ubiquitin chains, and at least in the case of yeast Rsp5, the isolated HECT domain is sufficient to synthesize such chains [49]. Our data support a model in which the ability to conjugate K63 chains to a viral or cellular substrate in the immediate vicinity of the emerging bud is central to the ability to stimulate virus release.

In our previous study, the unique potency of NEDD4-2s in the ΔPTAPP rescue assay did depend on its C2 domain being truncated, and was not shared by several other NEDD4 family members with intact C2 domains [36]. One possible explanation for these observations was that the natural truncation of the C2 domain in NEDD4-2s relieves an autoinhibition, which would be consistent with a study showing that the catalytic activity of a subset of C2-WW-HECT E3s is regulated through an inhibitory interaction between their C2 and HECT domains [42]. On the other hand, the residual C2 domain of NEDD4-2s was essential for activity in the ΔPTAPP rescue assay [36]. This finding raised the possibility that the C2 domain remnant of NEDD4-2s, which corresponds to β-strands 7 and 8 of the intact domain [42], constitutes a functional domain on its own that plays an active role in the rescue of HIV-1 budding. The present study supports this notion by demonstrating that the residual C2 domain of NEDD4-2s is sufficient to transfer the ability to rescue HIV-1 budding defects to other NEDD4 family ubiquitin ligases, and even to a subset of isolated HECT domains.

Our previous results suggested that the C2 domain remnant of NEDD4-2s is required for activity in the ΔPTAPP rescue assay, because it mediates the association of the ubiquitin ligase with HIV-1 Gag [36]. In support of this concept, we now show that other NEDD4 family members, and some isolated HECT domains, associate with HIV-1 Gag if tagged with the residual C2 domain of NEDD4-2s. Additional strong support is provided by the fact that we were able to functionally replace the C2 domain remnant of NEDD4-2s with CypA, an entirely unrelated protein that has long been known to specifically interact with HIV-1 Gag [40], [41].

HECT E3s contain two broad functional regions: a large N-terminal region required for substrate recognition, and a C-terminal region (the HECT domain) which catalyzes the ubiquitination of bound substrates [52]. Apart from the C2 domain, the N-terminal regions of NEDD4 family members harbor multiple WW domains, which we previously found dispensable for the rescue of HIV-1 budding by NEDD4-2s [36]. In the present study, essentially the entire N-terminal substrate recognition portion of NEDD4-2s became dispensable in the presence of CypA, which served as a Gag-targeting module. The simplest interpretation of this result is that no substrate other than Gag needs to be recognized to stimulate virus release. However, if a transacting factor rather than Gag is the relevant substrate for ubiquitination as proposed [53], then the isolated catalytic HECT domain of NEDD4-2s must be sufficient to recognize that factor.

One potential transacting factor is ESCRT-I, because Sundquist and colleagues have demonstrated that the stimulation of HIV-1_ΔPTAPP_ release by NEDD4-2s depends on Tsg101/ESCRT-I [37]. Furthermore, these authors showed that NEDD4-2s overexpression induces the ubiquitination of ESCRT-I complexes, particularly of those that contain MVB12B. They also reported that a PPQY sequence in MVB12B, which constitutes a potential binding site for WW domains, contributes to the ubiquitination of MVB12B/ESCRT-I complexes by NEDD4-2s. Based on these results, it was suggested that NEDD4-2s-mediated ubiquitination may activate ESCRT-I to function in HIV-1 release [37]. If this hypothesis is correct, then our observations imply that NEDD4-2s must remain capable of recognizing ESCRT-I as a substrate even in the absence of its N-terminal substrate recognition domain.

It has also been suggested that NEDD4 family E3s interact through their HECT domains with as yet unknown components of the ESCRT pathway, because several NEDD4 family members, and the isolated HECT domain of WWP1, localized to aberrant endosomal class E compartments induced by dominant-negative VPS4 [54]. We have now observed that Rsp5, the single C2-WW-HECT E3 of *Saccharomyces cerevisiae*, can strongly stimulate HIV-1_ΔPTAPP_ release and Gag processing when its C2 domain is replaced. Thus, if an interaction with an ESCRT pathway component is required for activity in the ΔPTAPP rescue assay, such an interaction and the interfaces involved must be conserved between yeast and man. One reported interaction that potentially meets these criteria is that between NEDD4 and ALIX or their yeast homologues Rsp5 and Bro1 [55]. Notably, the protein regions involved in the interaction appear conserved, because yeast Rsp5 co-immunoprecipitated with mammalian ALIX [55]. However, the NEDD4-ALIX interaction may depend on WW domains [55], which are dispensable for the ability of NEDD4-2s to rescue HIV-1_ΔPTAPP_ [36].

In principle, ESCRT pathway components could also be recruited via ubiquitinated Gag, because the upstream ESCRT complexes each possess at least one component that binds ubiquitin [6], [56]. For instance, the human ESCRT-I components Tsg101 and VPS37A contain ubiquitin-binding domains [12]. It was also recently reported that the ESCRT-associated protein ALIX specifically binds to ubiquitin [57].However, at least the ubiquitin-binding activity of Tsg101 is not required for the rescue of HIV-1 budding by NEDD4-2s [37]. Also, there is evidence that the ubiquitination of Gag is dispensable, because the PPxY-dependent budding of a foamy virus Gag protein completely devoid of ubiquitin acceptors could be stimulated by catalytically active WWP1 [53]. However, in the latter case, Gag-associated WWP1 could have served as an alternative ubiquitin acceptor, since the enzyme is capable of auto-ubiquitination.

K63-linked ubiquitin chains are required for the transport of at least some cargo into MVB [56], and have also been implicated in the function of PPxY-type L domains [58], which act by recruiting NEDD4 family members [13]. Several NEDD4 family members have indeed been shown to preferentially synthesize K63-linked ubiquitin chains, including mammalian NEDD4-1 and ITCH [49], [59], [60], as well as yeast Rsp5 [49], [61]. In contrast, E6AP, another HECT domain E3, preferentially synthesizes K48-linked chains, which provide a signal for proteasomal degradation [59]. At least in the case of ITCH, chain type specificity is determined by the C lobe of the HECT domain [49]. For instance, the replacement of the C lobe of ITCH with that of HUWE1 considerably reduces the preference for the synthesis of K63 chains [49]. In the present study, we observed that an ITCH/HUWE1 C lobe chimera did not efficiently rescue HIV-1 budding. Interestingly, the C lobe chimera retained the ability to efficiently ubiquitinate Gag, but lacked the ability of the parental ITCH construct to induce the attachment of K63-linked ubiquitin chains to Gag. Taken together, these data indicate that the ability to synthesize K63 chains is crucial for the stimulation of HIV-1 budding.

Structural studies indicate that the conformations of K63- and K48-linked chains are markedly distinct. Specifically, K63-linked di- or tetraubiquitin chains exhibit an extended conformation in which functionally important surface hydrophobic residues are constitutively exposed, whereas K48-linked chains can adopt a closed conformation in which these hydrophobic surface residues are sequestered [62], [63], [64]. It is thus likely that linkage-specific conformations provide a basis for the recruitment of distinct cellular recognition factors. Interestingly, it has recently emerged that K63-linked ubiquitin chains serve as specific signals for the ESCRT-dependent sorting of cargo into MVBs [56]. For instance, in the case of the yeast membrane protein Gap1, monoubiquitination is sufficient for its efficient endocytosis [65]. However, the presence of short K63-linked chains is required for the entry of Gap1 into the MVB pathway [65], suggesting preferential recognition of K63-linked chains by some component of the ESCRT machinery. The results presented here imply that HECT ubiquitin ligase-stimulated virus budding, which is also ESCRT-dependent [13], [66], is governed by the same type of ubiquitin modification.

## Materials and Methods

### Proviral constructs

HXBH10_ΔPTAPP_ is a mutant of HXBH10, a *vpu*-positive version of the infectious HXB2 proviral clone of HIV-1, with an in-frame deletion of codons 7 through 11 of p6 [44]. The Z_WT_ variant of HXBH10 encodes a chimeric Gag precursor that has NCp1p6 replaced by a leucine zipper dimerization domain [44].

### Expression vectors

Plasmids expressing NEDD4-2s, N_Δ1-31_ (residues 32–834 of NEDD4-2s), or WWP1s (residues 110–922 of WWP1) with an N-terminal FLAG tag have been previously described, and are based on the mammalian expression vector pBJ5 [36]. Vectors expressing CypA-HA/FLAG-NEDD4-2s fusion proteins were created using an overlap extension technique [67]. First, DNA fragments with overlapping ends were amplified in separate PCR reactions, using previously described vectors encoding CypA-HA and FLAG-tagged NEDD4-2s truncation mutants as templates [36], [68]. The two fragments were then recombined in a second PCR reaction as described [67], and inserted into pBJ5. The resulting Cyp-N_Δ1-31_, Cyp-N_Δ1-275_, and Cyp-N_ΔHECT_ constructs encode CypA followed by HA and FLAG epitopes, which in turn are followed by NEDD4-2s residues 32–834, 276–834, and 432–834, respectively. Vectors expressing CypA-HA/FLAG fused to the isolated HECT domains of SMURF1 (residues 366–757), E6AP (residues 462–852), or HERC6 (residues 637–1014) were made in an analogous manner using Cyp-N_Δ1-31_ and cDNA clones encoding SMURF1 (KIAA1625), E6AP (BC009271), or HERC6 (BC042047) as templates. Overlap extension PCR was also used to generate pBJ5-based vectors expressing FLAG-tagged residues 1–73, 1–110, or 1–245 of NEDD4-2s fused to residues 383–922 of WWP1 (yielding N_1-73_/WWP1, N_1-110_/WWP1, and N_1-245_/WWP1), using previously described vectors expressing FLAG-NEDD4-2s and FLAG-WWP1s as templates [36]. Furthermore, overlap extension PCR was used to generate pBJ5-based vectors expressing FLAG-tagged residues 1–37 of NEDD4-2s fused to residues 142–862 of ITCH (yielding N_1-37_/ITCH), residues 138–809 of yeast Rsp5 (yielding N_1-37_/Rsp5), residues 366–757 of SMURF1 (yielding N_1-37_/SMURF1_HECT_), residues 1207–1606 of HECW1 (yielding N_1-37_/HECW1_HECT_), residues 1162–1572 of HECW2 (yielding N_1-37_/HECW2_HECT_), residues 462–852 of E6AP (yielding N_1-37_/E6AP_HECT_), or residues 637–1014 of HERC6 (yielding N_1-37_/HERC6_HECT_). The templates used included previously published plasmids [36], [49], [69] and cDNA clones KIAA1625 (SMURF1), KIAA0322 (HECW1), KIAA1301 (HECW2), BC009271 (E6AP), and BC042047 (HERC6). The cDNA clones were provided by the Kazusa DNA Research Institute or purchased from Open Biosystems. Of note, each HECT domain construct includes the N-terminal H1′ helix of the HECT domain [70]. An expression vector for full-length Rsp5 with an N-terminal FLAG tag was generated using standard PCR. Finally, overlap extension PCR was used to generate pBJ5-based vectors expressing FLAG-tagged residues 1-245 of NEDD4-2s fused to residues 142-862 of WT ITCH (NM_031483), or to versions of ITCH in which the C lobe of the HECT domain has been replaced with the corresponding region of E6AP or HUWE1 [49].

### Assays for viral particle production and Gag ubiquitination

293T cells (1.2×10^6^) were seeded into T-25 tissue culture flasks 24 hrs prior to transfection. A calcium phosphate precipitation technique was used to transfect cells with HXBH10_ΔPTAPP_ (between 0.5 and 1 µg) or Z_WT_ proviral DNA (2 µg), along with expression vectors encoding the indicated E3 constructs (between 1 and 6 µg) or empty vector. Total DNA transfected was normalized to 8 µg by the addition of carrier DNA (pTZ18U). At 24 hrs post-transfection, cell culture supernatants were removed and clarified by low-speed centrifugation and passage through a 0.45 µm filter. Clarified supernatants were layered on to 20% sucrose, and viral particles were separated using high-speed centrifugation (27,000 rpm, 2 h, 4°C). Cells were lysed using 1 x RIPA buffer with protease inhibitors. Virus pellets and cell lysates were analyzed by SDS-PAGE and Western blotting. The anti-HIV CA antibody 183-H12-5C was used to detect Gag, Gag cleavage products, and Gag-ubiquitin conjugates. Ectopically expressed ubiquitin ligase constructs were detected using anti-FLAG (M2; Sigma) or anti-HA antibodies (HA.11; Covance). The K63-linkage specific antibodies Apu3 and HWA4C4 were purchased from Millipore. Western blots were quantitated with the ImageJ software.

### Accession numbers

NEDD4-2s: BC000621; WWP1: BC036065; ITCH: NM_031483; SMURF1: NM_020429; HECW1: NM_015022; HECW2: NM_020760; E6AP: NM_130838; HERC6: BC042047; HUWE1: NM_031407; CypA: NM_021130.
